# New Approach for the Identification of Isobaric and Isomeric Metabolites

**DOI:** 10.1021/acs.analchem.2c04962

**Published:** 2023-04-29

**Authors:** Ahmed Ben Faleh, Stephan Warnke, Teun Van Wieringen, Ali H. Abikhodr, Thomas R. Rizzo

**Affiliations:** Laboratoire de Chimie Physique Moléculaire, École Polytechnique Fédérale de Lausanne, EPFL SB ISIC LCPM, CH-1025 Lausanne, Switzerland

## Abstract

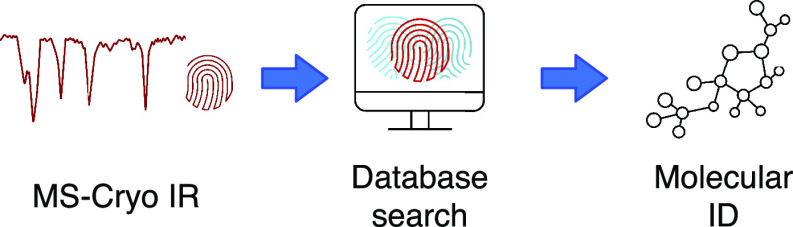

The structural elucidation of metabolite molecules is important in many branches of the life sciences. However, the isomeric and isobaric complexity of metabolites makes their identification extremely challenging, and analytical standards are often required to confirm the presence of a particular compound in a sample. We present here an approach to overcome these challenges using high-resolution ion mobility spectrometry in combination with cryogenic vibrational spectroscopy for the rapid separation and identification of metabolite isomers and isobars. Ion mobility can separate isomeric metabolites in tens of milliseconds, and cryogenic IR spectroscopy provides highly structured IR fingerprints for unambiguous molecular identification. Moreover, our approach allows one to identify metabolite isomers automatically by comparing their IR fingerprints with those previously recorded in a database, obviating the need for a recurrent introduction of analytical standards. We demonstrate the principle of this approach by constructing a database composed of IR fingerprints of eight isomeric/isobaric metabolites and use it for the identification of these isomers present in mixtures. Moreover, we show how our fast IR fingerprinting technology allows to probe the IR fingerprints of molecules within just a few seconds as they elute from an LC column. This approach has the potential to greatly improve metabolomics workflows in terms of accuracy, speed, and cost.

## Introduction

Metabolites are small-molecule products of biochemical reactions that are involved in essential cellular functions.^1−4^ Their analysis has proven to be important in a wide range of fields, including^4,5^ human health^5^ and nutrition,^5,6^ mammalian toxicology,^7^ plant chemistry,^8,9^ food quality,^10^ environmental sciences,^11,12^ microbial analysis,^13,14^ anti-doping,^15,16^ and clinical disease diagnostics.^17^ Many different techniques have been employed to identify and characterize metabolites. The most common are nuclear magnetic resonance (NMR) and liquid chromatography/tandem mass spectrometry (LC–MS/MS).^18−20^ While NMR offers unique capabilities in terms of molecular identification, it lacks the sensitivity to detect low-abundance species in complex biological samples. Conversely, LC–MS offers excellent sensitivity but provides limited structural information, which makes the identification of isobaric and isomeric metabolites difficult. Moreover, the limited transferability of MS/MS spectra between different instruments, the long LC retention times, and the need for continuous calibration restricts the throughput of LC–MS as well as the confidence in structural assignment based on these methods.^21,22^

Over the last 15 years, ion mobility spectrometry (IMS) has developed into a promising, commercially available technology for the analysis of isobars and isomers. It allows for orders of magnitude faster separation of metabolites (i.e., milliseconds versus minutes) and when properly calibrated provides collisional cross section (CCS) values, which contain information about the three-dimensional structure of the analytes.^15,16,23,24^ In addition, the recent advent of high-resolution IMS technologies such as trapped ion mobility spectrometry (TIMS),^25^ cyclic IMS (cIMS),^25^ and structures for lossless ion manipulations (SLIM)^26^ has enabled separation of isomeric species with the slightest of structural differences. Nevertheless, a CCS is not an intrinsic property of a molecule and requires precise and continuous calibration of the instrument, especially under high-resolution conditions where the error in the measured values can be in the same range as the difference between isomeric species.^26,27^ Moreover, identifying a complex molecular structure based on a CCS alone is tenuous, given the difficulty of calculating CCS values with sufficient accuracy to distinguish subtly different isomers.

Gas-phase vibrational spectroscopy has been gaining interest as an alternative approach to provide information on the molecular structure of metabolites.^28−30^ As opposed to experiments performed in the condensed phase, gas-phase infrared (IR) spectroscopy is performed on isolated molecules within the vacuum environment of a mass spectrometer. It allows the investigation of molecules isolated from a complex mixture and free from interaction with their surroundings while maintaining the sensitivity of mass spectrometry. During the past decades, infrared multiphoton dissociation (IRMPD) at room temperature has been used as a vibrational spectroscopic technique to characterize the molecular structure of small compounds such as amino acids, nucleotides, neurotransmitters, peptides, and glycans.^31−36^ In combination with computational models, IR spectroscopy can be a powerful tool to identify molecular structures.^37^ However, the limited resolution available in room-temperature IRMPD can, for example, impede identification of isomers.

Cryogenic IR spectroscopy offers a solution to this problem, as the lower temperature affords higher resolution, which facilitates distinguishing subtly different isomers. One way of implementing cryogenic IR spectroscopy inside a mass spectrometer is to use messenger tagging, in which an inert gas molecule that is transparent to IR light (e.g., N_2_) condenses onto collisionally cooled ions.^38^ Upon absorption of a single photon, vibrational energy is redistributed and the tag is dissociated from the parent ion. The absorption is detected in the mass spectrum by observing a decrease in the intensity of the messenger-tagged ion and an increase in the intensity of the untagged ions. This approach also provides the possibility of multiplexed spectral acquisition for ions of different *m*/*z*.

When analyzing mixtures of isomeric molecules, however, a separation step must precede spectral acquisition. While LC has been used to separate metabolite isomers prior to performing IRMPD-type experiments, this was either done offline by fraction collection^29^ or using stop-flow methods.^39^

We have previously reported the use of high-resolution IMS for isomer separation in combination with cryogenic IR spectroscopy for the identification of glycans present in mixtures.^40−46^ In this approach, the highly structured IR spectra represent unique molecular fingerprints that are stored in a database and used for identification. While high-resolution IMS allows us to rapidly separate isomers in the gas phase, cryogenic IR spectroscopic analysis of mobility-separated ions provides unambiguous identification. Moreover, recent advances in instrumentation allow us to record an IR fingerprint in less than 10 s using a turn-key, commercially available IR light source.^45^ In addition to the unmatched structural specificity, IR fingerprint identification requires neither continuous data calibration nor continuous use of analytical standards while benefiting from the sensitivity and selectivity of mass spectrometry. Once an IR fingerprint is stored in the database, it can be reproduced over time across different laboratories.

We report here proof-of-principle experiments combining high-resolution IMS with messenger-tagging IR spectroscopy for the fast identification of metabolite isobars and isomers. Eight isobaric metabolites from different origins were analyzed in this work. Kaempferol, quercitrin, naringenin, and luteoloside are antioxidants found in fruits, vegetables, and medicinal plants.^47^ They are known to mediate inflammation and have anticarcinogenic properties while being significantly less toxic to normal cells than conventional chemotherapy agents.^48−52^ Estradiol glucuronides are conjugated metabolites of estradiol, which is an estrogen steroid hormone. The two estradiol glucuronide isomers considered in this work are estradiol-3-β-D-glucuronide and estradiol-17-β-D-glucuronide. These two isomers result from the preferential glucuronidation by different enzymes and can have different biological activities prior to their elimination from the human body.^53^ We demonstrate a database approach that allows one to rapidly separate and assign isomeric metabolites based on their IR fingerprints.

## Methods

### Instrumentation

The experiments reported here were performed using a home-built instrument combining ultrahigh-resolution traveling wave (TW) IMS based on SLIM^54−56^ with cryogenic messenger-tagging IR spectroscopy. While the SLIM ultrahigh-resolution IMS module is used to separate isomeric molecules, their identification is based on their IR fingerprint. The details of the apparatus have been described previously.^45^ In brief, molecular ions produced by nano-electrospray are transferred into vacuum using a heated (130 °C) stainless-steel capillary and are guided through a dual ion funnel assembly toward the SLIM IMS region, which is held at a 2.2 mbar pressure of N_2_. Ions are loaded into a storage section within the SLIM device, where they are accumulated for most of the instrument duty-cycle time (typically 200 ms for the current experiments).^57^ Ion packets of 0.5–1.5 ms in duration are then released into the SLIM separation region, where they are transported by the TW potentials along a total drift path of ∼10.4 m, where they are separated according to their drift time, which is determined by their CCS. The SLIM device allows increasing the resolution by switching the electrodes at the exit of the drift path, routing the ions back toward the entrance of the separation region for additional cycles. In this way, we can achieve a resolving power of greater than 1000 after 18 separation cycles, corresponding to ∼190 m drift path. In addition to the ultrahigh resolving power, the high peak capacity afforded by the long single-pass path length that the SLIM technology offers provides a unique way to analyze samples with high isobaric/isomeric complexity on very short timescales.

Mobility-separated ions are then guided through differential pumping stages and loaded into a cryogenic ion trap, where we perform messenger-tagging IR spectroscopy.^38^ Prior to the arrival of the ions in the trap, a short pulse of buffer gas composed of a He:N_2_ mixture (80:20) is introduced and cooled to cryogenic temperatures (45 °K) by a closed-cycle cryostat (Sumitomo, Japan). Upon collisions with the cold buffer gas, ions form weakly bound clusters with N_2_ molecules, which serve as the messenger tag. During the ∼50 ms trapping time, the tagged ions are irradiated by a continuous-wave mid-IR laser (IPG Photonics, USA). When the frequency of an incident photon is in resonance with that of a molecular vibration, the photon is absorbed and its energy is redistributed throughout the molecule *via* intramolecular vibrational energy redistribution, causing the weakly bound N_2_ molecules to dissociate from the analyte ion. Ions can be directed toward the exit of the trap either by the electric field generated by a constant DC potential gradient along the trap (as applied in this work) or by application of a traveling potential wave that propagates toward the trap exit. After each instrument cycle, the trapped ions are released toward a time-of-flight mass spectrometer (Tofwerk, CH), where their mass-to-charge ratio is measured. An IR spectral fingerprint of the analyte ion is obtained by recording the TOF signal corresponding to the tagged molecules divided by the sum of the signals corresponding to tagged and untagged molecules as a function of the laser wavenumber in the range of 3300–3750 cm^–1^. Functional groups probed within this range correspond to OH and NH oscillators in a variety of different hydrogen-bonding arrangements, and as demonstrated below, there are a sufficient number of bands in this region to distinguish the set of molecules chosen for this study. While our currently employed laser source is limited to this range, this is not a fundamental limit to the technique, as we have previously measured IR spectra using messenger-tagging spectroscopy in the 5–10 μm region.^58^

Given a 200 ms cycle time for IMS, we obtain 5 data points in the IR spectrum per second. Depending upon the degree of averaging, an entire IR spectrum in the data reported here required 7–60 s to acquire.

### Automated Data Analysis *via* Principal Component Analysis (PCA) and Clustering

Our proposed workflow involves measuring IR fingerprints of metabolites separated from complex samples and comparing them to a database that is constructed using analytical standards. For routine analysis, spectral comparison is done automatically using a pattern recognition algorithm based on PCA and clustering. PCA is frequently used in exploratory data analysis and applied to various areas of analytical chemistry^59^ to reduce the dimensionality of large data sets and enable the implementation of predictive models. It allows the identification of a smaller number of dimensions, referred to as the principal components, which are sufficient to describe the original data set. The classification of IR fingerprints and their assignment to the reference molecules is done using an algorithm based on the clustering function in the “Scikit-learn” software Python library.^60^ A function based on the “soft K-means”^61,62^ method was implemented to determine the probability for the assignment of each vibrational spectrum to a spectrum in our database. In brief, soft K-means clustering allows each data point in PCA space to belong to multiple clusters of database spectra at the same time with different probabilities. This allows one to assess the probability of assigning a data point (i.e., spectrum) to a given cluster of database spectra and hence of identifying the molecule. Once an IR fingerprint is translated to PCA space, the probability of its assignment to a given predefined cluster is determined by its distance to the centroids of all the clusters. The closer the point is to a given cluster centroid relative to others, the higher the probability of its assignment to that cluster.^61^

### IR Fingerprint Deconvolution of Non-IMS Separated Isomers

In cases where isomers cannot be separated by IMS, the measured IR fingerprint will be the sum of the overlapping components and will not correspond to a single database entry. In this case, we perform IR fingerprint deconvolution using the *fminsearch* function in MATLAB, implemented in a custom-built algorithm. The *fminsearch* function iteratively minimizes the root-mean-square deviation (RMSD) between the IR fingerprint of the composite spectrum and that of a synthetic spectrum composed by combining different ratios of reference IR fingerprints. In this way, the algorithm returns the identity of the individual isomer components present in the mixture. We have recently reported the application of this algorithm to mixtures of human-milk oligosaccharides.^63^

### Materials

All metabolite standards were purchased from Sigma-Aldrich and Cayman Chemical. For nano-electrospray ionization, 1–10 μM solutions of the analytes were prepared in ethanol solutions for the reference material. The analytes present in the mixtures had a concentration of 1–2 μM. In-house prepared borosilicate glass emitters were used to inject samples into the instrument. In positive ion mode experiments, all molecules were analyzed in their singly sodiated form. In negative ion mode, all molecules were analyzed in their singly deprotonated form. All gases used were of 99.9999% purity.

### Sample Preparation for Parsley Flavonoid Extracts

Freeze-dried parsley (3 g) was extracted for 1 h in 100 mL of MeOH/H_2_O (80/20). Solids were subsequently filtered off using a Whatman filter. The filtrate was evaporated under a flow of air while heating to 40 °C. The evaporation residue was redissolved in 10 mL of boiling H_2_O. The sample was stored overnight at 8 °C and washed with hexane (2× 5 mL), and the flavonoid glycosides were extracted with *n*-ButOH (3× 4 mL). A 2 mL aliquot of *n*-ButOH solution was taken, and the solvent was evaporated under a flow of air. The residue was redissolved in 1 mL of MeOH/H_2_O (30/70) before analysis.

### Liquid Chromatographic Separation of Flavonoid Extracts

Separation was performed on a Waters Acquity 2.1 using a 50 mm UPLC BEH C18 1.7 μm column. A 10 μL aliquot was injected for analysis. Elution started with a linear gradient at a flow rate of 0.2 mL/min of 70% solvent A (MeOH/water 10/90 + 0.1% formic acid) and 30% solvent B (MeOH/water 90/10 + 0.1% formic acid) to 40% solvent A and 60% solvent B over the course of 25 min. The eluent was kept at 40% solvent A and 60% solvent B for 5 min. In 0.1 min, the eluent was changed back to the initial composition and the column was re-equilibrated for 4.9 min.

## Results and Discussion

### Building the Database for Metabolite Isobars/Isomers

**Figure 1:**
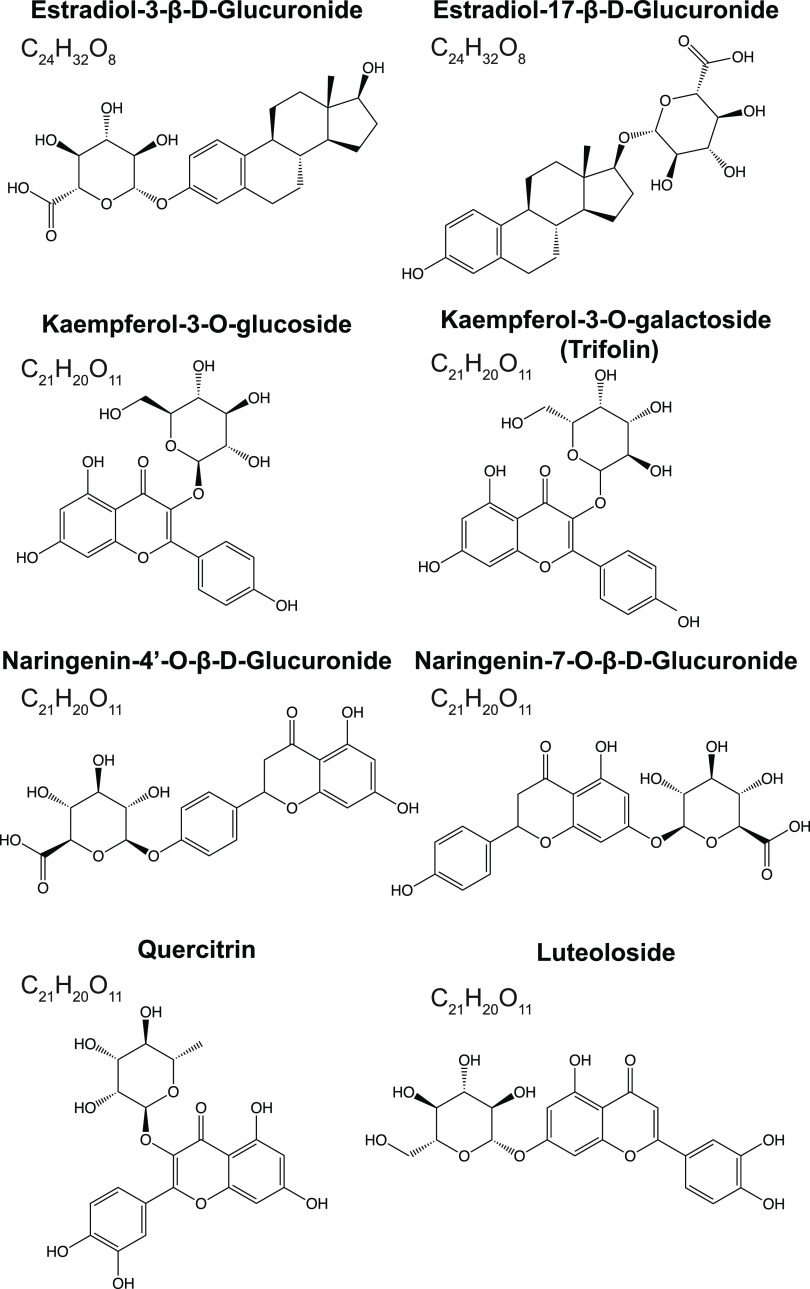
Structures of the metabolite isomers/isobars chosen for this study, all of which have a nominal mass of 448 Da.

The first part of this work was aimed at constructing an IR fingerprint database for a set of eight isobaric and isomeric metabolites (Figure 1) chosen for this proof-of-principle study. It includes two estrogen metabolite positional isomers, estradiol-3-β-D-glucuronide and estradiol-17-β-D-glucuronide. The remaining six isomeric metabolites are flavonoids originating from a variety of plants. Kaempferol-3-*O*-glucoside and kaempferol-3-*O*-galactoside have isomeric monosaccharides attached to the kaempferol core. Naringenin-4′-*O*-β-glucuronide and naringenin-7-*O*-β-glucuronide are positional isomers of each other. Quercitrin and luteoloside differ in their core as well as in the attached glycan. The structures of the eight metabolites are shown in Figure 1.

**Figure 2:**
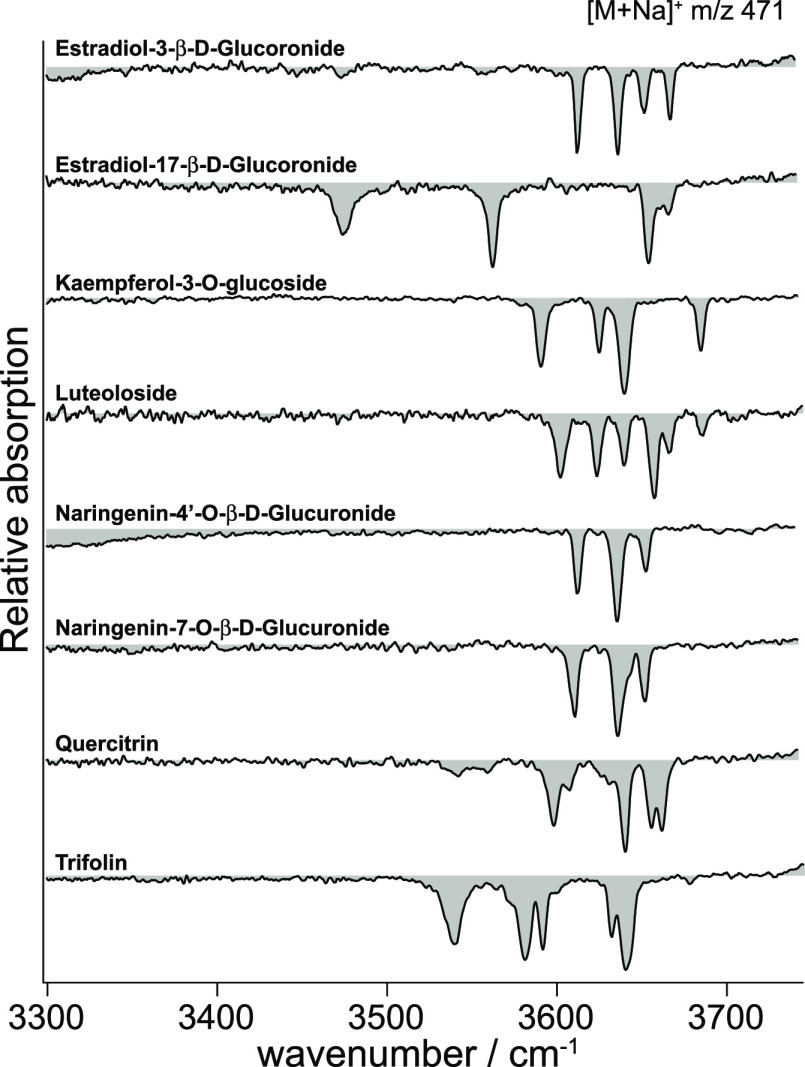
IR fingerprint spectra of the eight isomeric/isobaric metabolites in positive ion mode, recorded to be stored in the fingerprint database.

To build the initial IR spectral fingerprint database, the metabolite standards were first analyzed separately. The IR fingerprints of singly sodiated species were recorded in positive ion mode and are shown in Figure 2. Each absorption peak was oversampled during the acquisition to ensure an optimum signal-to-noise ratio and achieve a maximum spectral resolution.

We observe several well-resolved absorption bands originating from “free” or weakly hydrogen-bonded OH oscillators in the region between 3550 and 3700 cm^–1^ as well as more strongly hydrogen-bonded OH oscillators below 3550 cm^–1^. Although the structures of the metabolites are in some cases extremely similar, each spectrum is highly structured and unique, which makes them ideal fingerprints. We also observe that for the current set of metabolites, the region between 3500 and 3700 cm^–1^ would be sufficient for positive identification, as it includes most of the absorption bands. Spectra of the negatively charged (singly deprotonated) species were also recorded and feature similarly unique absorption bands (see Figure S1, Supporting Information).

### Rapid Acquisition and Automatic Fingerprint Comparison

**Figure 3:**
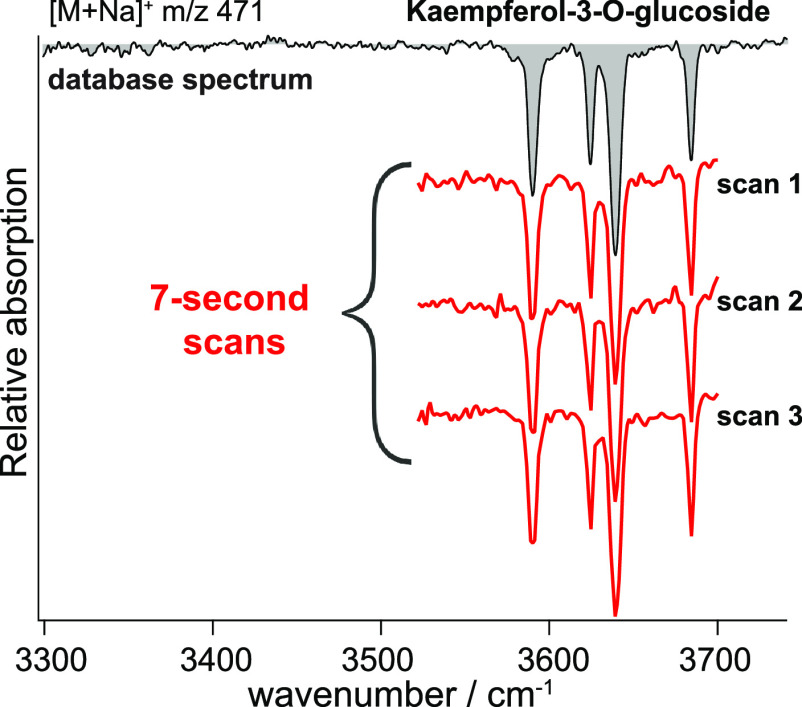
Rapid spectral acquisition: database spectrum (gray) acquired in 60 s compared to three replicate spectra acquired within 7 s each (red).

The database spectra shown in Figure 2 were each recorded in 60 s, which already represents a significant improvement compared to typical acquisition times of tens of minutes. Our current instrument features high signal stability and together with the use of a continuous-wave laser with stable output power allows for a considerable increase in the signal-to-noise ratio. This in turn reduces the amount of signal averaging required, drastically decreasing the acquisition times. However, to incorporate into routine-analytical workflows in metabolomics, IR spectral acquisition times should match typical LC elution times of only a few seconds. By shortening the wavelength scan range and down-sampling the recorded spectra, we can obtain reproducible fingerprints in as little as 7 s without losing the information that makes each spectrum a unique identifier. Figure 3 shows a comparison between the oversampled IR fingerprint (gray) of kaempferol-3-*O*-glucoside, recorded in 60 s, and three replicates for the fingerprint of the same molecule recorded in only 7 s (red). The excellent reproducibility of the individual measurements ensures that all the absorption bands in the scanned region are conserved and well resolved. A comparison between the database IR fingerprints of the eight metabolite isobars/isomers with those obtained using our rapid-scan method is shown in Figure S2. In all cases, the rapid scans are in excellent agreement with the corresponding database spectra.

**Figure 4:**
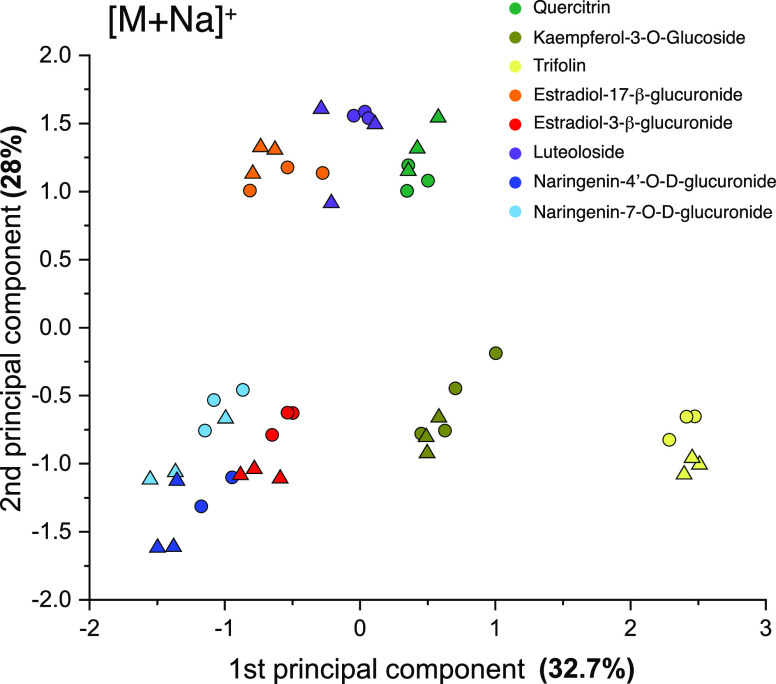
First and second components of a PCA performed on the database IR fingerprints (colored circles) and the IR fingerprints obtained by fast acquisition (colored triangles).

While it is clear by visual comparison that the spectra obtained using rapid scans contain sufficiently specific information to identify a given molecule, we tested our more rigorous approach using an algorithm based on PCA and clustering to automatically assign rapidly obtained spectra to those comprising our database. Figure 4 shows the results of this approach in the two-dimensional space corresponding to the first and second principal components (for simplicity, only two of eight PCA components are shown). Database spectra, of which multiple were previously recorded for each standard, are translated to PCA space, and each circle in Figure 4 corresponds to a given spectrum. Different colors represent the different species to which each data point corresponds, and these colors are automatically assigned by the clustering algorithm using eight PCA dimensions. After supplying the IR fingerprints recorded using our rapid-scan method to the algorithm, it automatically assigns each spectrum (represented by colored triangles in Figure 4) to its corresponding group, thereby successfully identifying each analyte. It is important to note that while different groups might not appear separated in the space spanned by the first and second principal components, they are unambiguously assigned using all eight principal components. The variability captured by the different principal components is displayed in Tables S1 and S2 for positive and negative ions, respectively. What is perhaps more helpful is to see the highest probability for the assignment of a particular spectrum to a given database spectrum, shown in Table S3, which is determined using the soft K-means algorithm by the distance of a particular IR fingerprint in PCA space to the centroid of an assigned cluster. Because of the unique and highly structured nature of the cryogenic IR spectra, the probability of a measured spectrum being assigned to a particular database spectrum is in most cases greater of 0.95.

The same PCA and clustering procedure was applied to data obtained for negatively charged ions [M – H]^−^ (shown in Figure S3). Also here, the algorithm successfully groups and thereby detects the IR fingerprints that belong to the same molecule. This approach has the potential to simplify data analysis considerably, as it requires no user input and can reliably assign IR fingerprints to their corresponding molecules in a fast, automated manner.

### Application to Metabolite Isomer Mixtures and IR Fingerprint Deconvolution

**Figure 5:**
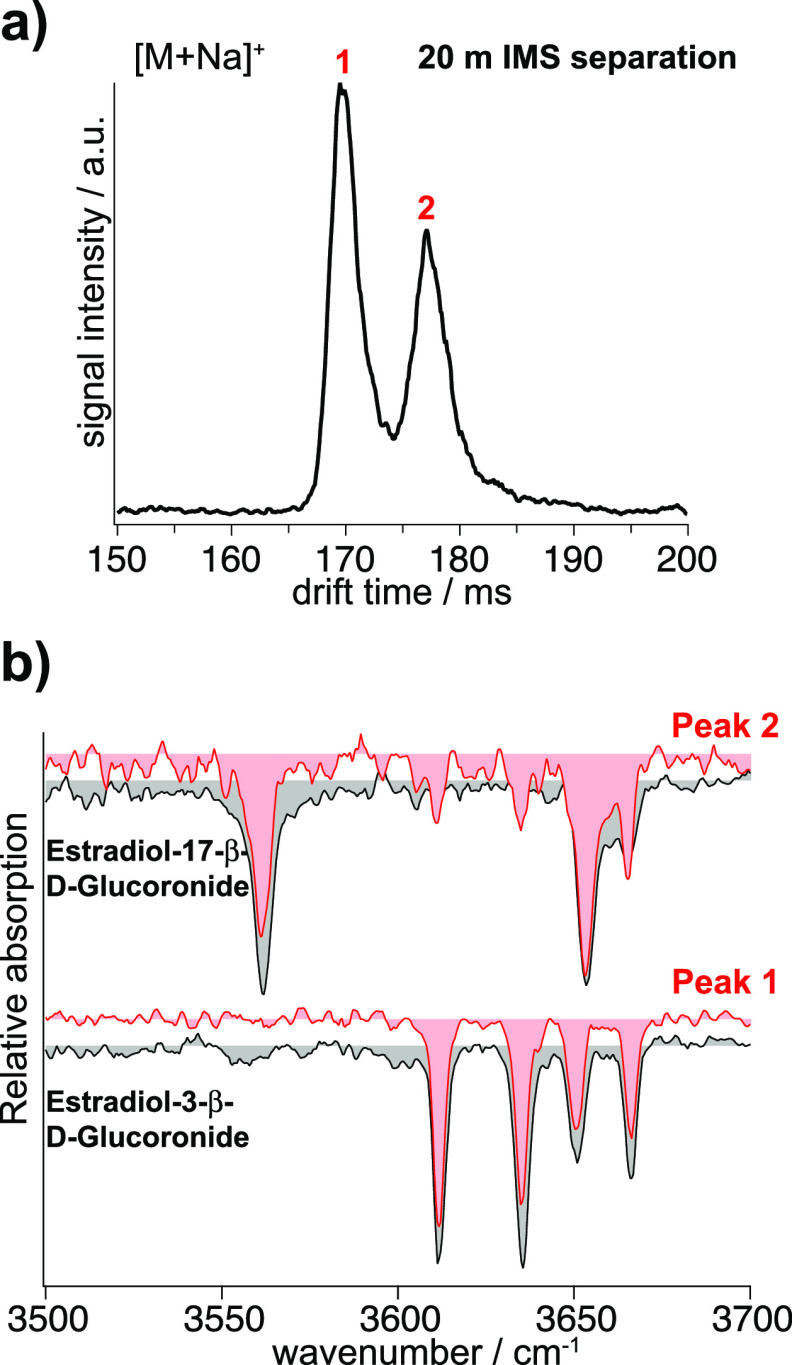
(a) Arrival time distribution of the mixture of the estradiol glucuronide isomers after 20 m separation. (b) IR fingerprints of the drift peaks in the ATD of the metabolite isomers (red), each recorded within 10 s, and their best-matching IR fingerprints from the database (gray).

We highlight the capabilities of our new approach by identifying the components of two different isomeric mixtures. The first contains the two estradiol glucuronide isomers shown in Figure 1. The solution was equimolar with a final concentration of ∼5 μM for each isomer. The arrival time distribution obtained after two separation cycles in the SLIM IMS device (20 m drift path) is shown in Figure 5a. We observe two mobility peaks, one per isomer present in the mixture. After mobility separation, IR fingerprints were recorded using a 10 s rapid acquisition scheme. A comparison between the fingerprints obtained from the mixture and the database entries for the estradiol glucuronide isomers is shown in Figure 5b. It is clear by visual comparison of the IR fingerprints that the first mobility peak can be assigned to estradiol-3-β-D-glucuronide and the second mobility peak to estradiol-17-β-D-glucuronide. The soft K-means algorithm makes these same assignments with average probabilities of 0.86 and 0.98, respectively (see Figure S4 and Table S4). In Table S4, we include probabilities for assignment to each of the clusters, and we do so for three different scans of the same spectrum. One can see that in the case of the spectrum associated with peak 1 in the arrival time distribution, if the signal-to-noise ratio is not sufficiently high, the algorithm returns a non-negligible probability of assignment to naringenin-4′-*O*-D-glucuronide rather than estradiol-3-β-glucuronide. This comes from the similarity of the spectra of these two species. Nonetheless, the assignment remains clear (and correct).

While it is tempting to use the intensities of the individual peaks observed in the arrival time distribution to extract quantitative information, care has to be taken that ionization efficiencies of individual compounds are accounted for, as in all MS-based quantification. The assumption of similar ionization efficiency for chemically similar compounds may lead to erroneous values. In addition, matrix effects during ionization may affect one isomer differently than another. A careful calibration procedure may be applied for such arrival time distributions to yield quantitative results.

**Figure 6:**
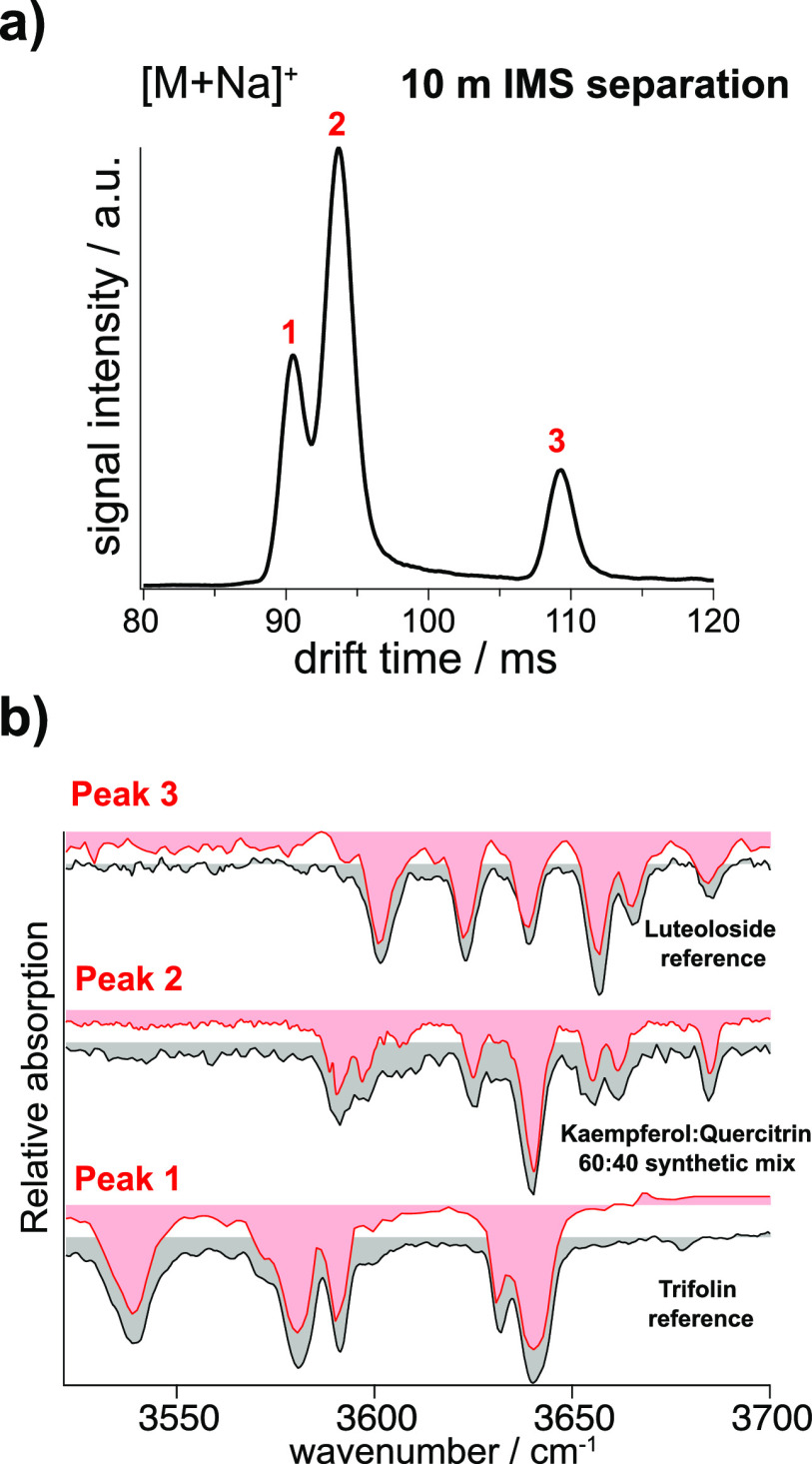
(a) Arrival time distribution of the mixture of four metabolite isomers after 10 m separation. (b) IR fingerprints of the drift peaks in the ATD of the metabolite isomers (red) and their matching IR fingerprints from the initial database for peaks 1 and 3 and a synthetic mixture resulting from the deconvolution algorithm for peak 2 (black).

The second isomeric metabolite mixture was composed of kaempferol-3-*O*-glucoside, kaempferol-3-*O*-galactoside (trifolin), quercitrin, and luteoloside. The solution was equimolar with a final concentration of ∼10 μM for each isomer. The ATD of this mixture, displayed in Figure 6a, exhibits three distinct mobility peaks. As the mixture is composed of four isomers, two of them must overlap. Using our IR fingerprinting method, we can assign peaks 1 and 3 to trifolin and luteoloside, respectively, based on the spectra displayed in Figure 6b. While the assignment is clear by visual comparison, it was further confirmed by our PCA and clustering algorithm, which gives confidence scores of 98 and 99%, respectively. The spectrum obtained for the second drift peak contains features from the two co-eluting isomers quercitrin and kaempferol-3-*O*-glucoside. While most features from the quercitrin IR fingerprint can be easily identified, the absorption at 3680 cm^–1^ and other details in the spectrum indicate the presence of kaempferol-3-*O*-glucoside. Using the deconvolution algorithm described under Methods, the spectrum of the species represented by the second drift peak can be reproduced by a 60:40 mixture of the database spectra for kaempferol-3-*O*-glucoside and quercitrin, respectively. While the algorithm for doing this has been tested previously for its ability to deliver quantitative results,^63^ we use it here only for a qualitative description of the isomeric content.

This example illustrates the analytical power of IR fingerprinting, even when no separation is possible. In the case of the set of eight metabolites used for this proof-of-principle study, IR fingerprinting allows the confident identification of all isomers present, even though high-resolution IMS cannot separate all of them. Furthermore, since the IR fingerprinting technology we use is incorporated within a mass spectrometer, it offers sensitivity comparable to that of classical LC–MS workflows.

### Online LC-IR Fingerprinting of Flavonoids from Herbs

Parsley finds application in traditional medicine as treatment against a variety of pathological conditions,^64,65^ and it is speculated that an abundance of flavonoids (1 mg/1 g fresh weight) is responsible for its pharmacological activity.^66^ A metabolite extract from fresh parsley leaves represents a complex mixture, and direct injection for analysis can result in suppression of signals from low-abundance species through charge competition or matrix effects during ionization. To cope with these effects, we perform LC separation prior to ionization and acquire IR fingerprints of the eluting analyte online within just a few seconds.

**Figure 7:**
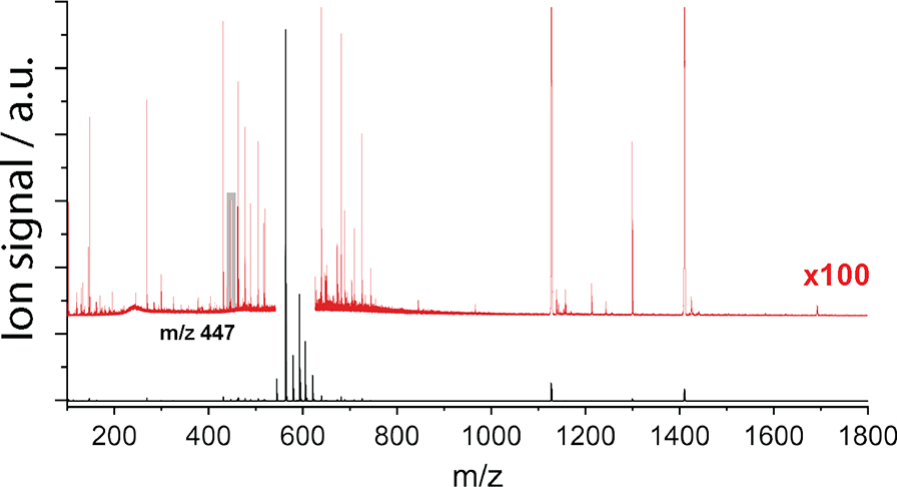
Mass spectrum of the parsley metabolite extract directly injected (i.e., without LC separation) and measured in negative ion mode. The signal corresponding in *m*/*z* to the isomeric compounds investigated in this work is highlighted in gray.

**Figure 8:**
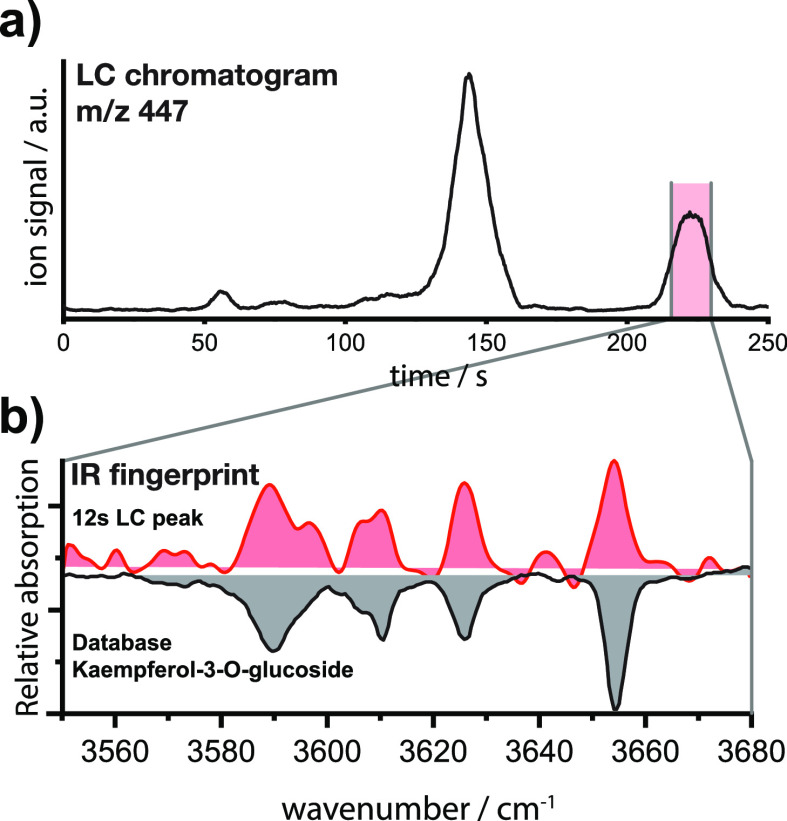
(a) LC retention time of ions with *m*/*z* 447 from the parsley metabolite extract. (b) IR fingerprint of the second LC feature, acquired during its 12 s of elution from the column (red), and the database IR fingerprint of kaempferol-3-*O*-glucoside as [M – H]^−^ species for comparison (gray).

A mass spectrum of the parsley metabolite extract in negative ion mode is displayed in Figure 7, and the signal that corresponds to the flavonoid glycosides investigated in this work at *m*/*z* 447 is highlighted in gray. Figure 8a shows the retention times of these ions and two features at 140 and 220 s, respectively, can clearly be distinguished. The IR spectrum of the second feature at 220 s, acquired while eluting from the column, is shown in Figure 8b (in red). The wavenumber range of 3550 to 3680 cm^–1^ was scanned within 12 s, which resulted in 60 data points over this range.

The spectrum features several well-resolved absorption bands and matches the database IR fingerprint of kaempferol-3-*O*-glucoside (shown in gray) in peak positions and intensities. We can therefore confirm the presence of kaempferol-3-*O*-glucoside with high certainty in the metabolite extract of parsley. This is further confirmed by our PCA and clustering algorithm, which assigns the spectrum to kaempferol-3-*O*-glucoside with a 90% certainty. The IR fingerprint of the first eluent at 140 s is not yet present in our database, and therefore, further investigative work is required to identify this species. We estimate the concentration of kaempferol glucoside in the metabolite extract to be on the order of 10 nM, based on the signal response of the corresponding eluent in the LC chromatogram when compared to that from a sample that was spiked with an analytical standard of kaempferol glucoside at a concentration of 100 nM (see Figure S5 in the Supporting Information).

We found the lowest limit of detection to be on the order of 10 nM by assessing IR fingerprint signal quality and reproducibility of kaempferol glucoside ions in negative and positive ion modes. IR fingerprints obtained using 10 nM solutions are shown in Figure S6 in the Supporting Information and show good signal quality and reproducibility even at these low concentrations. This opens the door for application of IR fingerprinting in the analysis of metabolites present in body fluids, where molecules of interest are present in low abundance.

## Conclusions

We have used high-resolution IMS in combination with cryogenic IR spectroscopy for the identification of isomeric and isobaric metabolites. We created an initial database composed of the IR fingerprints of eight isomeric/isobaric metabolites including six isomeric flavonoids (kaempferol-3-*O*-glucoside, kaempferol-3-*O*-galactoside, naringenin-4′-*O*-β-glucuronide, naringenin-7-*O*-β-glucuronide, quercitrin, and luteoloside) as well as two estrogen positional isomers (estradiol-3-β-D-glucuronide and estradiol-17-β-D-glucuronide). The database includes IR fingerprints of both positively charged (singly sodiated) and negatively charged (singly deprotonated) metabolites. Using rapid fingerprinting, we are able to record highly reproducible IR fingerprints in as little as 7 s.

In the second part of this work, we demonstrated the ability of our approach to distinguish and identify metabolite isomers present in mixtures by (a) separating different isomers using high-resolution IMS and (b) identifying them by comparing their IR fingerprints to those in our previously recorded database. Using an algorithm based on PCA and machine learning clustering, we demonstrate the ability to automatically assign the observed IR fingerprints to their corresponding molecules with high confidence (>90%). When it is not possible to separate isomers by high-resolution IMS, we show that it is possible to deconvolute the spectrum of the isomeric mixture and identify its components using a custom algorithm based on iteratively minimizing the RMSD.

Another important result demonstrated here is that the speed at which we can measure an IR spectrum allows us to couple it with liquid chromatography in real time. It is important to note that unlike current LC–MS/MS workflows, the approach applied in this work does not require recurrent calibration with analytical standards. Once an IR spectrum is measured for a particular metabolite using a standard, this spectrum is put in a database and never needs to be measured again. Moreover, calibration of the laser may be checked at most once every several months in a standard and straightforward manner.

Our approach of combining either high-resolution IMS or liquid chromatography for isomer separation with cryogenic IR spectroscopy for identification, together with automated IR fingerprint identification and spectral deconvolution, has the potential to become a powerful new tool in the field of metabolomics.

## Supporting Information Available

IR fingerprint spectra of negatively charged metabolites; rapid-scan IR spectra compared to database; and details of PCA-based spectral assignments (PDF)
