# Supplementary material for: New Approach for the Identification of Isobaric and Isomeric Metabolites
Source: Anal Chem. 2023 Apr 29;95(18):7118–26. doi: 10.1021/acs.analchem.2c04962 (PMC10173252; doi:10.1021/acs.analchem.2c04962)
Supplement: Supplementary file 1 — ac2c04962_si_001.pdf [file ac2c04962_si_001.pdf]

SUPPORTING INFORMATION

A new approach for the identification of isobaric and isomeric metabolites

Ahmed Ben Faleh, Stephan Warnke, Teun van Wieringen, Ali H. Abikhodr, and Thomas R. Rizzo*

Laboratoire de Chimie Physique Moléculaire, École Polytechnique Fédérale de Lausanne, EPFL SB ISIC LCPM, CH-1025 Lausanne, Switzerland

*email: thomas.rizzo@epfl.ch

Table of Contents

Figure S1: IR fingerprint spectra of the eight metabolites in negative ion mode . . . . page S-2

Figure S2: Fast scan of IR fingerprints compared to their database equivalents . . . . page S-3

Table S1: Percent variability captured by PCA components (Positive ions) . . . . page S-3

Table S2: Percent variability captured by PCA components (Negative ions). . . . . page S-3

Table S3: Highest probabilities of spectral assignments . . . . page S-4

Figure S3: First and second components of PCA (Negative ions) . . . . page S-4

Figure S4: First and second component of the PCA (Positive ions) . . . . page S-5

Table S4: Confidence of assignments for isomeric mixture . . . . page S-5

Figure S5: LC retention times of molecules from the parsley extract . . . . page S-5

Figure S6: Replicate scans of the IR fingerprints of kaempferol-3-O glucoside . . . . page S-6

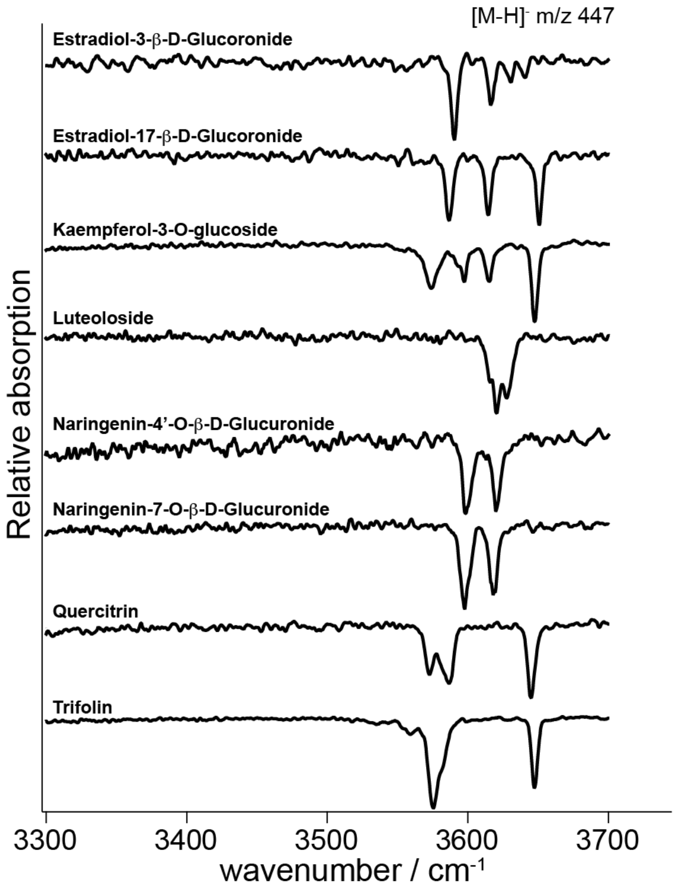

Figure S1: IR fingerprint spectra of the eight isomeric/isobaric metabolites in negative ion mode $[M-H]^-$, recorded to be stored in the fingerprint database.

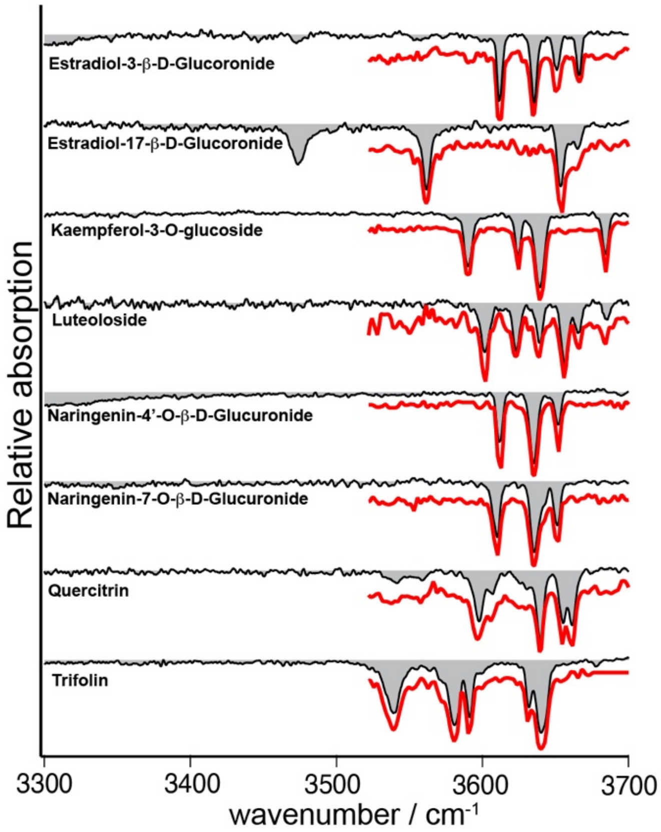

Figure S2: IR fingerprints of the eight isomeric/isobaric metabolites in the singly sodiated form $[M+Na]^+$ obtained within just a few seconds each (red), compared to their database equivalents (grey). The characteristic absorption features of each molecule are maintained in the spectra obtained during the rapid scans.

Principle component	1	2	3	4	5	6	7	8
% Variability	32.7	28	13.6	7.8	4	2.7	1.7	1.1

Table S1: Variability captured by PCA components (Positive ions)

Principle component	1	2	3	4	5	6	7	8
% Variability	44	14.3	10	7.5	4.1	3.1	1.9	1.7

Table S2: Variability captured by PCA components (Negative ions)

	fast scan 1	fast scan 2	fast scan 3
**Trifolin**	0.9996	0.9997	0.9968
**Kaempferol-3-O-Glucoside**	0.9965	0.9999	0.9918
**Quercitrin**	0.9997	0.9991	0.9889
**Luteoloside**	0.9986	0.9875	0.8160
**Estradiol-17-β-glucuronide**	0.9991	0.9894	0.9977
**Estradiol-3-β-glucuronide**	0.9482	0.8924	0.7094
**Naringenin-4'-O-D-glucuronide**	0.9837	0.9896	0.9573
**Naringenin-7-O-D-glucuronide**	0.9598	0.9937	0.9961

Table S3: The highest probabilities for the assignment of the rapid-scan spectra of Figure S2 to a cluster of database spectra based on the distance of the data to the centroids of the corresponding cluster in PCA space. Data for three replicate measurements are shown.

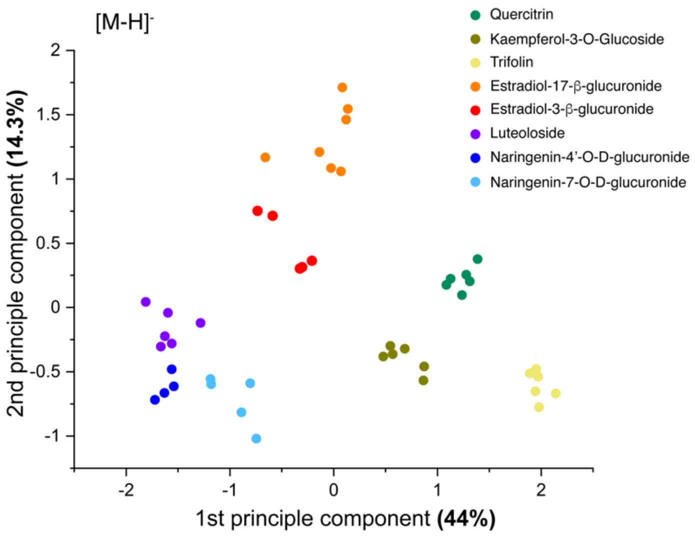

Figure S3: First and second components of a PCA performed on database IR fingerprints of singly deprotonated species [M-H]$^-$. Each group of spectra (different replicates), represented by the different colors, is detected automatically by the algorithm.

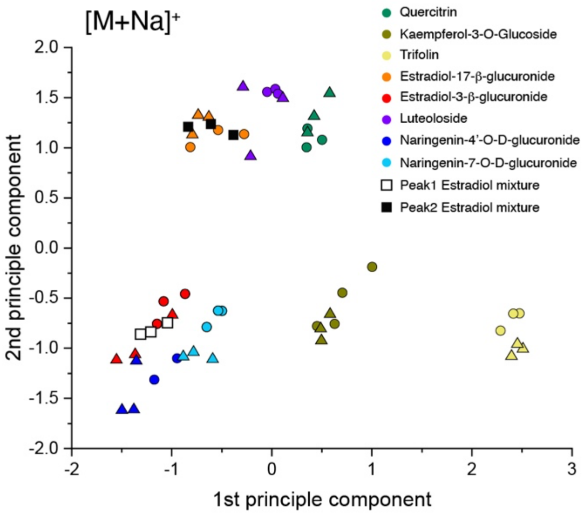

Figure S4: First and second component of the PCA performed on the IR fingerprints of the eight isomeric/isobaric molecules in positive ion mode (as displayed in Figure 5 of the main manuscript), including the data obtained from the first (open squares) and second (filled squares) mobility peaks of the first metabolite mixture composed of the two estradiol glucuronide isomers. Data for three replicate measurements is shown, each performed within 10 seconds. In each case, the individual spectra are successfully assigned to the correct group of database IR fingerprints in PCA space.

	**Peak1 scan1**	**Peak1 scan2**	**Peak1 scan3**	**Peak2 scan1**	**Peak2 scan2**	**Peak2 scan3**
**Trifolin**	0.00005	0.0016	0.0003	0.0015	0.0006	0.00006
**Kaempferol-3-O-Glucoside**	0.0001	0.0043	0.0008	0.0110	0.0037	0.0004
**Quercitrin**	0.0002	0.0086	0.0014	0.0011	0.0011	0.0002
**Luteoloside**	0.0002	0.0055	0.001	0.0182	0.0055	0.0005
**Estradiol-17-β-glucuronide**	0.0002	0.0008	0.1004	0.9499	0.9817	0.9979
**Estradiol-3-β-glucuronide**	0.9803	0.7207	0.8863	0.0077	0.0036	0.0005
**Naringenin-4'-O-D-glucuronide**	0.0161	0.221	0.0972	0.0031	0.0014	0.0002
**Naringenin-7-O-D-glucuronide**	0.0021	0.029	0.0113	0.0057	0.0024	0.0003

Table S4: Probabilities of assignment of IR fingerprints from peak 1 and 2 of Figure 5 to the respective clusters of IR fingerprints in PCA space.

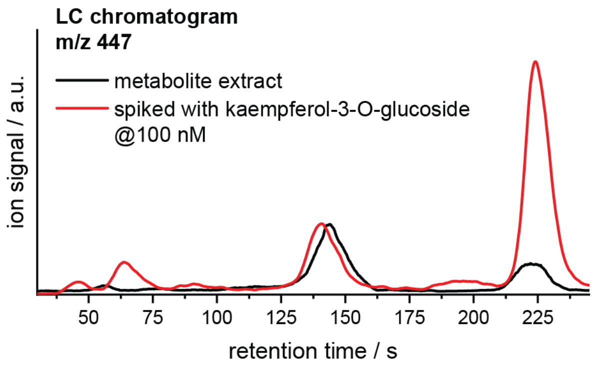

Figure S5: (Black trace) LC retention times of molecules from the parsley metabolite extract corresponding by mass to the eight isomeric/isobaric species investigated in this work. (Red trace) Data from the same metabolite extract, spiked with an analytical standard of kaempferol-3-O-glucoside at a concentration of 100 nM. The difference in signal intensity of the kaempferol glucoside feature at 220 s allows to estimate the concentration in the parsley sample to be on the order of 10 nM.

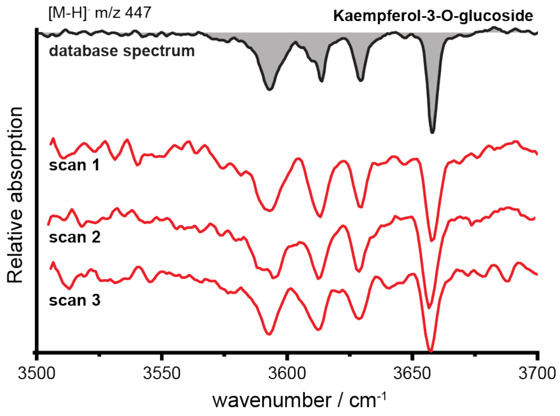

Figure S6: Three replicate scans of the IR fingerprints of kaempferol-3-O glucoside obtained from a 10 nM sample are shown in red. The corresponding database spectrum is shown above in gray. The signal quality of the data obtained from the low-concentration sample is sufficient for an unambiguous identification.
